# A simplified protocol for *in vitro* rearing of human body lice

**DOI:** 10.1051/parasite/2020007

**Published:** 2020-02-10

**Authors:** Jose E. Pietri, Ritesh Ray

**Affiliations:** 1 University of South Dakota, Sanford School of Medicine, Division of Basic Biomedical Sciences Vermillion 57069 SD USA

**Keywords:** Body lice, Rearing, Feeding, *In vitro*, Artificial, Method

## Abstract

Human body lice (*Pediculus humanus*) are neglected ectoparasites and pathogen vectors. Difficulties in raising and maintaining colonies of body lice in a laboratory setting remain a barrier to fundamental studies of physiology and vector-pathogen interactions in these insects. Several *in vivo* and *in vitro* rearing systems have been previously described and used by multiple research groups. However, these methods suffer from drawbacks that still complicate the rearing of body lice relative to many other commonly studied hematophagous insects. Here, a simplified protocol for raising and maintaining body lice *in vitro* using the commercially available Hemotek apparatus is described. This protocol draws from published methods for rearing body lice as well as other hematophagous insect species to further reduce labor, time, costs, and regulatory requirements typically associated with keeping human body lice in the laboratory. Using this protocol, the insects consistently fed on commercially available rabbit blood with little mortality, reached adulthood at a high rate, and produced a significant number of viable eggs, resulting in a 4.8-fold increase in population over a period of 40 days. The data suggest that the process described here can propagate modest populations for ongoing laboratory experiments and is a useful alternative to existing methods. The use and further optimization of *in vitro* rearing systems may facilitate dynamic studies of body lice by a wider range of investigators, enabling new progress in combating lice infestations, and louse-borne infections.

## Abstract (fr)

Les poux du corps de l’homme (*Pediculus humanus*) sont des ectoparasites et des vecteurs de pathogènes négligés. Les difficultés à élever et à maintenir des colonies de poux du corps en laboratoire restent un obstacle aux études fondamentales en physiologie et des interactions vecteur-pathogène chez ces insectes. Plusieurs systèmes d’élevage *in vivo* et *in vitro* ont déjà été décrits et utilisés par plusieurs groupes de recherche. Cependant, ces méthodes souffrent d’inconvénients qui compliquent encore l’élevage des poux du corps par rapport à de nombreux autres insectes hématophages couramment étudiés. Ici, un protocole simplifié pour élever et maintenir des poux du corps *in vitro* en utilisant l’appareil Hemotek, disponible dans le commerce, est décrit. Ce protocole s’inspire des méthodes publiées pour l’élevage des poux du corps ainsi que d’autres espèces d’insectes hématophages pour réduire le travail, le temps, les coûts et les exigences réglementaires généralement associées à la conservation des poux du corps dans le laboratoire. En utilisant ce protocole, les insectes se nourrissent constamment de sang de lapin disponible dans le commerce avec peu de mortalité, atteignent l’âge adulte avec un taux élevé et produisent un nombre important d’œufs viables, entraînant une augmentation de 4,8 fois de la population sur une période de 40 jours. Les données suggèrent que le processus décrit ici peut propager des populations modestes pour des expériences de laboratoire et est une alternative utile aux méthodes existantes. L’utilisation et l’optimisation des systèmes d’élevage *in vitro* peuvent faciliter les études dynamiques des poux du corps par un plus large éventail de chercheurs, permettant de nouveaux progrès dans la lutte contre les infestations par les poux et les infections transmises par les poux.

## Introduction

Human body lice, *Pediculus humanus humanus*, are ectoparasites that predominantly affect marginalized populations [5]. Among these populations, body lice are responsible for the transmission of bacterial pathogens that can cause serious illness including: *Bartonella quintana*, *Borrelia recurrentis*, and *Rickettsia prowazekii* [12] However, despite their clinical significance, body lice are largely neglected by the biomedical research community.

One factor that has limited basic laboratory research into the biology of body lice is the perceived difficulty associated with rearing the insects in a laboratory setting. Under natural conditions, both head and body lice have constant access to their host and thus take multiple daily blood meals [4, 30]. Unsurprisingly, mimicking this natural rhythm in the lab is problematic. As a result, early studies of human lice employed human volunteers as a blood source for colonies. One such method requires an individual to wear an improvised containment capsule harboring living lice directly on the body (e.g., strapped on the wrist or ankle) for a large portion of each day, allowing the insects to blood feed through a barrier *ad libitum* [27]. Similarly, a process in which a human volunteer undertakes daily blood feedings by placing lice directly on their body under careful watch for a single interval of time has also been used [8, 27]. While the approach of enlisting a human volunteer to raise lice is both a logistical burden and potential irritant to the host, it is still used to a limited extent to this day [26, 28].

Some human-derived populations of body lice were eventually adapted to feeding on rabbits daily or several times per week [9, 10]. Today, rabbits are commonly used to maintain colonies of body lice, as well as to perform toxicological studies and experimental infections with louse-borne pathogens [2, 6, 11, 13, 16, 17, 19, 28]. Though effective, this method requires cumbersome manipulations of vertebrate animals (e.g., shaving, restraining) and is dependent on approval by an Institutional Animal Care and Use Committee. In addition, costs and access to facilities required to maintain vertebrate animals for rearing lice may be prohibitive for some laboratories, and obtaining pathogen-free rabbits from commercial colonies can also be a hurdle.

A limited number of *in vitro* methods for feeding body lice have been described. For example, some groups have used improvised apparatuses to contain animal blood in a membrane bound capsule and warm it on a hot plate or circulating water bath [14, 15, 18, 20]. Others have employed commercially available blood feeding devices such as the Hemotek apparatus [24]. These approaches are less cumbersome than animal-based protocols and enable controlled experimental blood feeds, but in most cases they have proved insufficient to support colony development due to low feeding or high mortality rates. For example, in Kasai et al. two thirds of immature lice fed on an artificial system died before reaching adulthood [18], and in Mumcuoglu et al. less than 50% of adults fully engorged on an artificial system [20]. While Sangare et al. achieved high rates of engorgement and low mortality in adults fed using a Hemotek device, they did not examine the ability of nymphs to feed on the same system [24]. Nevertheless, several automated and manual adaptations of an *in vitro* rearing system conceived by one group have enabled the successful propagation of head and body lice colonies [28, 29, 31]. To our knowledge, these systems remain the only described options for rearing human lice without the use of vertebrates. Drawbacks of these systems include that they require constant exposure of the insects to the heated blood source, making it susceptible to contamination, as well as a fair amount of craftsmanship to assemble and maintain. In contrast, commercial devices such as the Hemotek can be purchased fully assembled with all necessary components included.

Here we describe a simplified alternative protocol for rearing body lice. This approach draws from previously described systems for feeding these insects as well as strategies used to maintain other hematophagous species. Notably, the protocol described here uses the commercially available Hemotek device to allow for both experimental blood feeds and the propagation of a colony with minimal effort and several key advantages over other methods.

## Materials and methods

### Body louse strain and rearing conditions

The body louse strain used in the present study is from a long-standing colony fed on live rabbits in Dr. Kostas Mumcuoglu’s lab at the Hebrew University of Jerusalem. This strain was originally derived from a colony at the London School of Tropical Medicine and Hygiene in 1984, and most likely originated from the initial adaptation of the Culpepper strain to a rabbit host [8]. The insects were shipped to Dr. Jose Pietri’s laboratory at the University of South Dakota and housed on small pieces of a cut cotton shirt in ventilated plastic jars at 30 ± 1 °C and 70–80% relative humidity.

### Blood source and preparation

**Figure 1:**
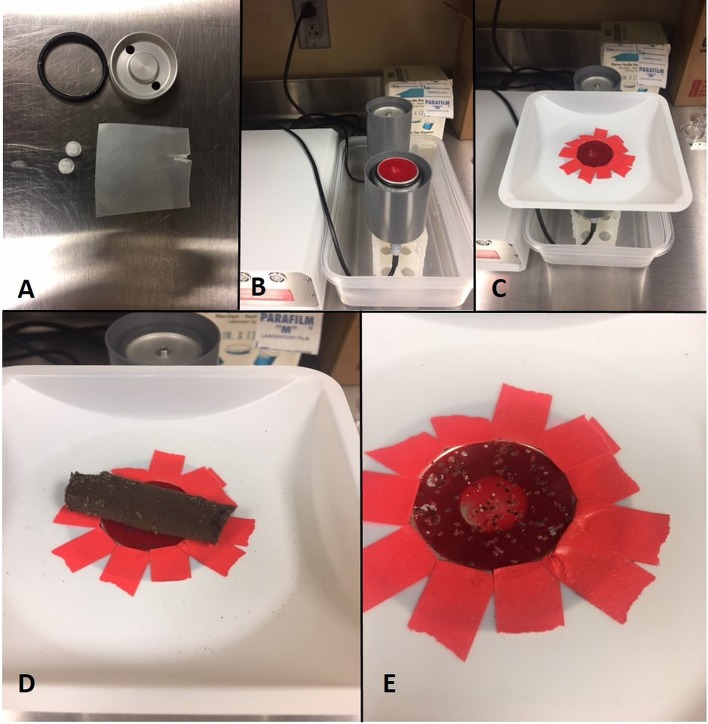
Visual of *in vitro* feeding process. (A) A 2 mL metal reservoir is assembled using Parafilm M, a rubber O-ring, and plastic plugs provided with the Hemotek apparatus. (B) The reservoir is filled with 2 mL of defibrinated rabbit blood using sterile technique and screwed into an inverted FU1 feeding unit. (C) A barrier cut from a plastic weighing boat is placed on top of the Parafilm membrane forming a gentle seal that prevents escape of the lice. (D) The rearing substrate harboring lice is placed directly on the feeding membrane after turning on the device to pre-warm for 2–3 min. (E) Within minutes, lice can be seen leaving the harborage and beginning to feed. By 30 min, feeding rates >90% are common. Many fed lice return to the harborage independently after engorging, but lice remaining on the membrane after feeding can be returned to the harborage by gently swabbing it over the membrane surface.

Aseptic, mechanically defibrinated rabbit blood was purchased from a commercial vendor (Hemostat Laboratories, Dixon, CA, USA). Sterility of the blood was maintained by only opening the bottle of blood inside a class II biosafety cabinet. As such, no preservatives or antibiotics were added. A Hemotek PS6 artificial membrane feeding system was used for *in vitro* blood feeding of the lice, according to the manufacturer’s general recommendations (Hemotek Ltd., Blackburn, UK, http://hemotek.co.uk/products/, Fig. 1A). In brief, a 2 mL metal blood reservoir (Hemotek product code R37P30) was disinfected by washing in 10% bleach, rinsing with deionized water, and spraying with 70% ethanol. Inside a biosafety cabinet, a small piece of Parafilm M membrane was thinly stretched over the metal reservoir and secured with a rubber O-ring (Hemotek product code OR37-25). Still inside the biosafety cabinet, 2 mL of rabbit blood were added to the reservoir, which was subsequently plugged with sterilized plastic stoppers (Hemotek product code PP5-250). The reservoir was removed from the biosafety cabinet and stored at 4 °C until the time of feeding on the same day that it was prepared.

### Blood feeding procedure and data collection

To begin the feeding, the prepared blood reservoir was removed from the refrigerator, gently swirled, and screwed into an FU1 feeder unit (Hemotek product code FU1-0) that had been inverted on a plastic conical tube rack and set to a temperature of 37 °C with a Hemotek thermometer probe (product code DTKFU1) (Fig. 1B–E). A barrier made from a disposable plastic weigh boat with a hole cut in the middle and taped to prevent sharp edges from puncturing the membrane was added to the reservoir. When this is placed onto the Parafilm surface it forms a gentle, reversible seal that prevents most lice from escaping the membrane surface. Although some small lice are able to escape the membrane surface by going underneath the weigh boat, these quickly fall into the groove of the FU1 feeder unit from which they cannot climb out and can be re-collected easily. Additional containment measures such as Vaseline barriers may be incorporated into the FU1 feeder or weigh boat as desired, but are not necessary. The reservoir was warmed for 2–3 min, and then cotton substrates harboring body lice were placed directly onto the membrane. Groups of lice were allowed to feed on the artificial system for 30 min each day and for 5 days each week (Monday–Friday) before returning to the incubator. Between each feed, the barrier was gently removed without rupturing the Parafilm and the blood reservoir was returned to storage at 4 °C. A fresh blood reservoir was prepared each week to prevent bacterial contamination and degradation of the blood from heating. To examine the ability of the artificial system to maintain and support the growth of a colony, daily blood feeding success and survival after each blood feed were measured for a subset of lice over a period of 30 days. We also measured the time to each molt, the number of eggs produced, and the hatch rate of the eggs produced. These experiments were initiated with three independent cohorts of ten newly emerged first instar nymphs.

## Results

### Feeding success and survival

**Figure 2:**
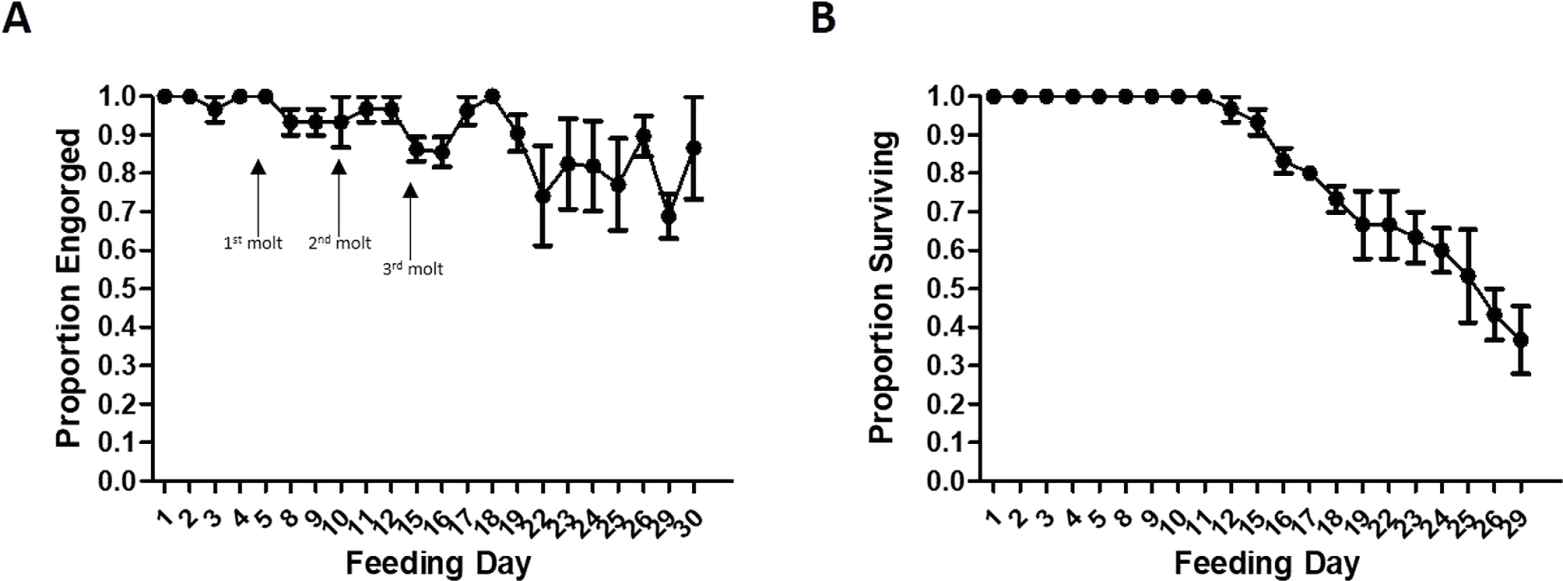
Feeding success and survival of lice fed on *in vitro* system. (A) The proportion of living lice engorging was measured after each 30 min feeding session. (B) The proportion of lice surviving each feeding session was measured on the subsequent feeding day. *N* = 3 biological replicates of 10 lice. Plots indicate mean ± SEM.

In each of three trials, feeding success and survival of lice nymphs was high throughout the entire time course (Fig. 2). In particular, for the first 11 days of each experiment, the proportion of lice feeding on each day was 90% or greater, and survival of the lice was 100%. After day 12 of feeding, a low level of mortality began to be observed and slowly increased over the subsequent days. This mortality correlated with increased variation in feeding success, which began to fluctuate and generally slightly decreased when the lice reached adulthood after approximately 15 days of feeding. Between day 15 and the termination of the experiments on day 30, feeding remained in the 68–100% range on average and mating of adults could be observed after feeding. Moreover, an average of 37% of the starting population survived for the entire 30 days. Although the experiments were stopped at this time, the results suggest that it may be possible to continue the feeding protocol for additional days in order to maximize colony growth.

### Life history

In each of the three trials, the development of the lice was highly synchronized (Table 1). In each trial, the first instar nymphs all molted to the second instar stage after the fifth day of feeding. Similarly, in each trial all second instar nymphs molted to the third instar after 10 or 11 days of feeding. An average of 80% of nymphs that began the feeding protocol reached adulthood and this took 15–17 days on our feeding regimen. The first eggs were observed after 18 days of feeding in the first trial and after 19 days of feeding in the second and third trials. Subsequently, a total of 152 eggs (average of 50.66 eggs/trial) were laid directly on cotton harborages over a period of 30 days. Egg hatching was first observed on day 26 of the experiment in each trial. In total, 143 of the 152 eggs hatched (94%), resulting in a population growth of 4.8-fold over 40 days. It is likely that egg production, like feeding, could have continued beyond the 30 day period of the experiments leading to a greater number of eggs laid. Nonetheless, the life history results to this point indicated with certainty that propagation of a colony is possible using our system.

**Table 1:** Life history traits of lice fed on *in vitro* system.

	Average days of feeding to second instar	Average days of feeding to third instar	Average days of feeding to adult	Eggs laid (by day 30)	% of eggs hatching
Trial 1 (*n* = 10)	5 (*n* = 10)	10.2 (*n* = 10)	15.25 (*n* = 8)	64 (*n* = 5 females)	96.8% (62/64)
Trial 2 (*n* = 10)	5 (*n* = 10)	10.1 (*n* = 10)	15.25 (*n* = 8)	32 (*n* = 4 females)	78.1% (25/32)
Trial 3 (*n* = 10)	5 (*n* = 10)	10.2 (*n* = 10)	15.75 (*n* = 8)	56 (*n* = 5 females)	100% (56/56)
Average	5 (*n* = 30)	10.17 (*n* = 30)	15. 42 (*n* = 24)	10.8/female	94% (143/152)

## Discussion

In this report, we describe a simplified protocol for rearing a colony of human body lice using a commercially available *in vitro* blood-feeding apparatus. This regimen provides logistical advantages over other commonly used systems and embodies several key advances in body lice rearing.

The simplicity of our protocol is similar to that of protocols used to feed and maintain colonies of non-fastidious hematophagous species, including many mosquitoes and bed bugs [1, 7, 21, 22]. By employing the Hemotek device, handling the blood and blood reservoir using sterile technique, and storing blood at 4 °C between feedings, only ~2 mL of blood are needed each week to feed a colony of hundreds of lice. However, the system is highly flexible and can be contracted or expanded to include 1–6 feeding units, each accommodating 100–200 lice. The low volume of blood required and the use of rabbit blood as opposed to that of humans or other vertebrate animals reduces costs to under $3 USD per week after the initial investment in the feeding apparatus (~$2000 USD including all components). Further, less than 3 h of time each week are required to maintain the colony, and most of this time is unsupervised feeding. In fact, inexperienced individuals can be trained to maintain lice on the Hemotek system in just a few minutes with no additional supplies or equipment.

A second advantage of the system we describe is that it represents the adaptation of body lice to an irregular feeding schedule on an *in vitro* system. While this has been achieved for *P. humanus* reared on rabbits, which are fed every 24–48 h, it is a unique property among artificial feeding approaches published for this species. A similar feeding schedule has been used to maintain colonies of the cat flea, *Ctenocephalides felis*, with a Hemotek device [3]. Much like body lice, cat fleas are normally persistent feeders that continually feed on their host and do not survive long without a blood meal. Therefore, the apparently successful adaptation of cat fleas to feeding only 5 days of the week served as inspiration for our own regimen. Although a multi-day period of starvation may reduce feeding success on the next day that a blood meal is offered, this effect is not drastic and feeding success quickly recovers in the following days (Fig. 2A). It is likely that the feeding schedule can be further adjusted to fit diverse needs, providing additional flexibility.

Lastly, eliminating the need for preservatives or antibiotics in the blood is another significant advance that may explain why appreciable population growth was observed even without continual feeding. The addition of penicillin/streptomycin to the blood meal does not appear to inhibit head lice reproduction *in vitro* [28, 29]. However, others have shown that doxycycline, erythromycin, azithromycin, and rifampicin can have detrimental effects on body lice by killing their endosymbionts [23, 24], as can preservatives such as EDTA through unknown means [20]. At the very least, it is unlikely that such additives provide any benefit to the lice and thus should be avoided if possible to minimize sub-lethal physiological alterations. At the same time, the sterility and integrity of the blood meal is critical to preventing mortality after feeding. Simple measures such as handling blood using sterile technique, storing the blood at 4 °C between feedings, and preparing a fresh blood reservoir weekly appear sufficient to maintain these parameters, but if discoloration of the blood is noticed, a fresh reservoir should always be prepared. Precautions such as storing the blood reservoir in a sterile container between feeds or disinfecting the Parafilm M membrane with ethanol prior to use may be useful for improving sterility and outcomes if contamination issues arise.

Ultimately, our data demonstrate that the system we describe is effective at propagating body lice from one generation to the next and is therefore a useful alternative to other systems. Although the exact growth kinetics of our entire colony have not been tracked, we have been able to sustain rearing of a largely synchronized colony of 500–700 individuals per generation, which has enabled biological replication of experimental work. It remains to be determined whether there are generational effects that will cause colony health to decline over time on the artificial system, as these were not measured in the present study. A similar concern is that stochastic variations in blood quality due to issues at the source could cause unexpected colony crashes, though these are also a concern with *in vivo* approaches. To date, there are few studies that compare the life history of human lice reared using different methods. *In vivo* rearing appears to yield better results in some strains [28], but other strains of body lice fed on rabbits have been reported to take as long as 21 days to reach adulthood, which is substantially delayed relative to our results [25].

Future work is needed to determine if other laboratory or field strains of human lice can be adapted to our protocol, or if alternative blood sources (e.g., human blood) can be incorporated. A notable caveat to our study is that we utilized a body louse strain that was already adapted to feeding on rabbits five times per week, and we adapted this strain to feeding on a similar regimen and blood source. However, at least one rabbit adapted body louse strain has been switched to human blood using another *in vitro* system [28]. The continued improvement of *in vitro* rearing systems for body lice and the adaptation of additional populations to these systems have the potential to facilitate novel, dynamic studies of a neglected ectoparasite of global importance.

## Conflicts of interest

The authors have no conflicts of interest to disclose.
